# Titanium dioxide nanoparticles (TiO_2_ NPs) promote growth and ameliorate salinity stress effects on essential oil profile and biochemical attributes of *Dracocephalum moldavica*

**DOI:** 10.1038/s41598-020-57794-1

**Published:** 2020-01-22

**Authors:** Gholamreza Gohari, Asghar Mohammadi, Ali Akbari, Sima Panahirad, Mohammad Reza Dadpour, Vasileios Fotopoulos, Seisuke Kimura

**Affiliations:** 1 Department of Horticultural Sciences, Faculty of Agriculture, University of Maragheh, Maragheh, Iran; 2 Solid Tumor Research Center, Cellular and Molecular Medicine Institute, Urmia University of Medical Sciences, Urmia, Iran; 3 Department of Horticultural Sciences, Faculty of Agriculture, University of Tabriz, Tabriz, Iran; 4 Department of Agricultural Sciences, Biotechnology and Food Science, Cyprus University of Technology Limassol, Limassol, Cyprus; 5 Department of Industrial Life Sciences, Kyoto Sangyo University, Kamigamo-Motoyama, Kita-Ku, Kyoto 603-8555 Japan; 6 Center for Ecological Evolutionary Developmental Biology, Kyoto Sangyo University, Kamigamo-Motoyama, Kita-Ku, Kyoto 603-8555 Japan

**Keywords:** Plant sciences, Materials science, Nanoscience and technology

## Abstract

Considering titanium dioxide nanoparticles (TiO_2_ NPs) role in plant growth and especially in plant tolerance against abiotic stress, a greenhouse experiment was carried out to evaluate TiO_2_ NPs effects (0, 50, 100 and 200 mg L^−1^) on agronomic traits of Moldavian balm (*Dracocephalum moldavica* L.) plants grown under different salinity levels (0, 50 and 100 mM NaCl). Results demonstrated that all agronomic traits were negatively affected under all salinity levels but application of 100 mg L^−1^ TiO_2_ NPs mitigated these negative effects. TiO_2_ NPs application on Moldavian balm grown under salt stress conditions improved all agronomic traits and increased antioxidant enzyme activity compared with plants grown under salinity without TiO_2_ NP treatment. The application of TiO_2_ NPs significantly lowered H_2_O_2_ concentration. In addition, highest essential oil content (1.19%) was obtained in 100 mg L^−1^ TiO_2_ NP-treated plants under control conditions. Comprehensive GC/MS analysis of essential oils showed that geranial, z-citral, geranyl acetate and geraniol were the dominant essential oil components. The highest amounts for geranial, geraniol and z-citral were obtained in 100 mg L^−1^ TiO_2_ NP-treated plants under control conditions. In conclusion, application of 100 mg L^−1^ TiO_2_ NPs could significantly ameliorate the salinity effects in Moldavian balm.

## Introduction

Moldavian balm (*Dracocephalum moldavica* L.), a perennial herb of the Lamiaceae family and native to central Asia, naturalized in central and eastern Europe and is cultivated around the world as a medicinal plant. Essential oils and extracts of Moldavian balm have been traditionally used as a painkiller for kidney complaints, toothache and colds. In addition, it has antimicrobial activities^1^, antirheumatic, antitumor, antimutagenic, antioxidant and antiseptic properties^2^. Aerial parts of Moldavian balm are important sources of monoterpene glycosides, trypanocidal terpenoids, rosmarinic acid and flavonoids^3^.

Salinity stress is considered as one of the main environmental factors limiting plant distribution in their natural habitats^4^. Soil salinity affects about 800 million hectares of arable land worldwide. Salinity stress causes major problems regarding plant growth, development and productivity, especially in arid and semi-arid regions of the world^5^ manifested as changes in morphological, physiological and biochemical characteristics of plants, ion toxicity (Na^+^ and Cl^−^), nutritional disorders and osmotic stress. These negative impacts significantly decrease plant yield under salinity stress conditions^6^. The tolerance mechanisms of plants to salinity stress are different in terms of osmotic regulation, CO_2_ assimilation, toxic ion uptake, ion compartmentation and/or exclusion, chlorophyll content, chlorophyll fluorescence, reactive oxygen species (ROS) generation, antioxidant defenses and photosynthetic electron transport^4,7^. Several studies have recently focused on new strategies to deal with salinity in order to minimize its negative effects^8,9^.

Nanotechnology is the study and application of small-sized materials (1–100 nm), a specific quality that makes these tiny entities unique. Thus, application of nanoparticles is one of the new strategies to improve growth and plant performance under salinity stress^10^. Titanium dioxide (TiO_2_) nanoparticles (NPs) lead to various profound effects on morphological, physiological and biochemical properties of some plant species. Lei *et al*.^11^ reported that the application of TiO_2_ NPs improved rubisco and antioxidant enzymes activities, photosynthetic rate and chlorophyll formation that subsequently caused enhanced crop yield. Latef *et al*.^12^ reported positive effect of TiO_2_ NPs on enhancement of plant growth, antioxidant enzyme activities, soluble sugars, amino acids and proline content in addition to a reduction in H_2_O_2_ and MDA contents in broad bean plants under saline conditions. Khan^13^ reported mitigation of salt stress by TiO_2_ NP application in tomato by improving agronomic traits, leaf chlorophyll content, phenolics and antioxidant capacity, antioxidant enzyme activities and yield. TiO_2_ could be considered as a stimulant for plants that activates different defense mechanisms involved in plant tolerance against various abiotic stress factors^11^. These effects might vary under different environmental conditions or in diverse plant species and based on the applied concentrations^14,15^. Similar to other NPs, the size, shape and concentration of TiO_2_ NPs have very important roles in their application. On the contrary, several reports have presented the negative and toxic effects of high concentration of TiO_2_ in plants that varied between plant tissues, growth stages and plant species based on concentrations and properties of nanoparticles^14,16^. Therefore, concentration, size, method of treatment application, uptake by plants, properties, reactivity and translocation of NPs into different tissues could determine NP interference with various metabolic activities that lead to toxic impacts^17,18^. Furthermore, surface area of NPs, their reactive nature and tendency to aggregate are other possible reasons for their toxicological effects^19^. High concentration of TiO_2_ NPs mainly results in the elevated production of oxygen reactive species (ROS), followed by chlorophyll degradation and cellular toxicity^20^. In addition, cell wall and plasma membrane damage due due to high NP concentration result in NP interaction with various cellular process^18,21^. In fact, the toxic effect of TiO_2_ NPs is dose- and time- dependent and putative mechanisms leading to toxicity are oxidative stress through ROS over-production, cell wall damage and lipid peroxidation. TiO_2_ NP toxicity also depends on species, particle size and exposure condition^21^. The toxic effects of TiO_2_ NPs have been reported in barley^14^, tobacco^21^, onion^22^, wheat^23^ and spinach^24^ plants. The discovery of their widespread uses in agriculture and plant science is still under debate^16^. Thus, the present study tried to investigate beneficial and toxicological impacts of different concentrations of TiO_2_ NPs in nutrient solution on key morphophysiological and biochemical characteristics as well as essential oil profile in Moldavian balm, an aromatic and medicinal plant, grown under salinity stress conditions. In addition, the uptake and aggregation of TiO_2_ NPs in the plant root was investigated by epifluorescence microscopy.

## Materials and Methods

### Preparation of TiO_2_ NPs

TiO_2_ NPs were synthesized according to the protocol previously reported^25^. Briefly, desired amount of titanium isopropoxide was hydrolyzed and stirred vigorously at 4 °C to produce white precipitate of TiO(OH)_2_. The obtained precipitate was washed three times with distilled water and dissolved in nitric acid to obtain clear and homogeneous titanyl nitrate [TiO(NO_3_)_2_] solution. For the synthesis of TiO_2_, titanyl nitrate and urea solution with 1:1 molar ratio was kept in a 250 mL beaker and put into a muffle furnace at 400 °C. After 2 h, the solid product was collected as TiO_2_ NPs and stored in vacuum oven until usage.

### Chemicals and Instruments

All chemicals and solvents were purchased from Merck and Sigma-Aldrich (Germany) and used without further purification. A Win-Bomem spectrometer, version 3.04 Galactic Industries Corporation over the range of 400–4000 cm^−1^ was used to obtain Fourier transform infrared (FT-IR) spectra. The synthesized TiO_2_ NPs were coated with a thin layer of gold and visualized using a scanning electron microscopy/energy dispersive X-ray spectroscopy (SEM/EDX) instrument, VWGA3 TESCAN (20.0 KV). For recording the Transmission electron microscopy (TEM) images, so-called TiO_2_ NPs were dispersed in distilled water and used a Zeiss EM-90 operating at 80 kV tension. Wide angle X-ray diffraction (XRD) profiles of TiO_2_ NPs were collected by using a Bruker D8 Advance diffractometer with wavelength, λ = 0.154059 nm (Cu Kα) at 30 keV.

### Epifluorescence microscopy

In order to study the uptake of TiO_2_ NPs, epifluorescence microscopy was employed in the treated plants. Plant materials were stained with 0.1% auramine O solution in water for 10 min. Samples were observed using an Olympus BX51 (Olympus optical Co., Ltd. Tokyo, Japan). Fluorescence microscope was equipped with the catadioptric lenses UMP lan FL-BDP and the BXRFA (Olympus optical Co., Ltd. Tokyo, Japan) fluorescence illuminator^26,27^. Image (z-stack) acquisition was performed using an Evolution MP cooled CCD (Media Cybernetics, USA) high-resolution digital camera. For this purpose, series of consecutive images from different focal planes of the sample were taken and then superimposed automatically to improve the depth of focus using ImageJ 1.41 software (http://rsbweb.nih.gov/ij/) in accordance with Dadpour *et al*.^28^. Outputs from the z-stack acquisitions were trimmed and saved as TIFF-format images.

### Experimental site, plant materials and Tio_2_ NPs treatments

The study was conducted at the research greenhouse of Department of Horticultural Sciences, University of Maragheh, Maragheh, East Azerbaijan Province, Iran (longitude 46°16′E, latitude 37°23′N, altitude 1485 m) as a factorial experiment in a completely randomized design (CRD). The experiment consisted of twelve treatments (each with three independent biological replications), three levels of salinity ((0, 50 and 100 mM NaCl) and four levels of TiO_2_ NPs (0, 50, 100 and 200 mg L^−1^). The seeds of Moldavian balm (*Dracocephalum moldavica* L.) were purchased from Pakanbazr Company, Isfahan, Iran. Regarding seed preparation, surface sterilization of the seeds was done with 1% (w/v) sodium hypochlorite (NaOCl) for 5 min, then washed three times with distilled water and finally soaked in distilled water for 10 min. The seeds were wetted with tap water and let to germinate for a week. Then, in each pot, eight plants were hydroponically grown in growth medium containing cocopite and perlite (2:1 ratio). Plants were irrigated daily with quarter-strength Hoagland solution with some modification^29^. After three weeks, salinity stress was imposed (eight-leaf stage), applied daily (in combination with quarter-strength Hoagland solution) and continued up to plant harvest (prolonged stress ≈ two months after applying salt stress) for the establishment of salinity effects on plant agronomic parameters. TiO_2_ NPs were added three times (three continuous days) to quarter-strength Hoagland solution two weeks after salinity stress application. Control plants were irrigated daily with quarter-strength Hoagland solution until harvest and treated with 0 mM NaCl and 0 mg L^−1^ TiO_2_ NPs.

### Agronomic parameters

Plant agronomic traits including plant height, shoot and leaf fresh and dry weights and leaf number were recorded at the harvest stage. For this purpose, five plants from each treatment were randomly sampled to measure the above traits. For fresh and dry weights, five samples were individually weighed for fresh weight and then kept in the oven (70 °C, 72 h) for dry weight measurements.

### Chlorophyll a, b and carotenoid content

Chlorophyll (Chl) and carotenoids amounts were achieved by extracting 0.2 g of fresh leaves in 0.5 mL acetone (3% v/v). After centrifuging (10000 rpm, 10 min) and obtaining the supernatant, absorption was recorded at 645 nm (Chl *b*), 663 nm (Chl *a*) and 470 nm (carotenoids) by UV-Vis spectrophotometry (UV-1800 Shimadzu, Japan). The youngest and fully expended leaves (from growing point) were used for measurements. Photosynthetic pigment contents (Chl *a*, *b* and carotenoids) were calculated from the following equations as described by Sharma *et al*.^30^:

$${\rm{Chlorophyll}}\,a=(19/3\ast {\rm{A}}663\,-\,0/86\ast {\rm{A}}645)\,{\rm{V}}/100{\rm{W}}$$

$${\rm{Chlorophyll}}\,b=(19/3\ast {\rm{A}}645\,-\,3/6\ast {\rm{A}}663){\rm{V}}/100{\rm{W}}$$

$${\rm{Carotenoids}}=100({\rm{A}}470)\,-\,3/27({\rm{mg}}\,{\rm{chl}}\,b)\,/227$$

Note: V = Solution volume of the filtrate, A = Light absorption in wavelengths 663, 645 and 470 nm and W = Sample fresh weight (g).

### Chlorophyll fluorescence

Chlorophyll fluorescence parameters (^Fv^/_Fm_, ^Fv^/_Fo_ and Y(ll)) were measured using dual-pam-100 chlorophyll fluorometer (Heinz Walz, Effeltrich, Germany) after adaption of *D. moldavica* in the dark for 20 min^31^.

### Hydrogen peroxide (H_2_O_2_) content

H_2_O_2_ content of Moldavian balm leaves was measured according to Sinha *et al*.^32^. Briefly, fresh leaves (0.2 g) were homogenized with 5 mL trichloroacetic acid (0.1% w/v) in an ice bath and then centrifuged (12000 rpm, 15 min). At that time, 0.5 mL of the supernatant was added to 0.5 mL potassium phosphate buffer (pH 6.8, 10 mM) and 1 mL potassium iodide (KI) (1 M). Finally, the absorbance of the mixture was recorded at 390 nm. H_2_O_2_ (µmol g^−1^ FW) content was estimated by standard calibration curve previously made by various H_2_O_2_ concentrations.

### Antioxidant enzyme activity assays

Young and fully expanded leaves were collected to assay antioxidant enzymes activities. For this purpose, samples were collected in an ice bucket and brought to the laboratory. All steps of enzyme extraction were carried out at 4 °C as follows: 0.5 g of the homogenized leaves were extracted with potassium phosphate buffer (pH 6.8, 10 mM) containing 1% polyvinylpyrrolidone (PVP) using magnetic stirrer for 10 min. The homogenate was centrifuged (6000 rpm, 20 min) and the supernatant was used for the assay of catalase (CAT), ascorbate peroxidase (APX), superoxide dismutase (SOD) and guaiacol peroxidase (GP) enzyme activities.

In order to determine CAT activity, the mixture of 0.5 mL potassium phosphate buffer, 4.5 mL H_2_O_2_ (3%) and 50 µL crude enzyme extract in a quartz cuvette was assayed using a UV-Vis spectrophotometer (UV-1800 Shimadzu, Japan) at 240 nm for 120 s^33^.

SOD activity was measured based on the method described by Sun *et al*.^34^ with slight modifications. The reaction mixture consisted of 2.5 mL potassium phosphate buffer, 0.2 mL methionine (0.2 M), 0.1 mL EDTA (3 mM), nitro blue tetrazolium (NBT), 1 mL distilled water, 0.1 mL NaCa_3_ (1.5 M), 0.1 mL riboflavin and 50 µL enzyme extract illuminating in glass tubes. The unilluminated mixtures were used as blanks. Test tubes were exposed to light by immersing in a beaker 2/3 filled with clean water, maintained at 27 °C. The increase in absorbance due to formazan formation was recorded at 560 nm. One unit of SOD was defined as the amount of enzyme that inhibited the rate of nitro blue tetrazolium reduction by 50%.

Considering APX activity^35^, the reaction mixture consisted of 250 µL potassium phosphate buffer, 250 µL ascorbate (1 mM), 250 µL EDTA (0.4 mM), 190 µL distilled water, 250 µL H_2_O_2_ (10 mM) and 0.5 mL enzyme extract. The changes in absorbance of samples at 290 nm, demonstrating enzymatic activity, were recorded and the extinction coefficient was considered as 2.8 cm^−1^ mmol^−1^.

The assay mixture for the estimation of GP activity comprised of 1 mL potassium phosphate buffer, 250 µL EDTA, 1 mL guaiacol (5 mM), 1 mL H_2_O_2_ (15 mM) and 50 µL enzyme extract. The rate of change in absorbance at 470 nm was determined according to Tang and Newton^36^.

### Essential oil extraction and profiling

The essential oils were extracted from 50 g air-dried powdered aerial parts of plants by the hydro-distillation technique and heated by heating jacket at 100 °C for 2 h in an all-glass Clevenger type apparatus, according to procedures outlined in the European pharmacopeia. The collected crude essential oils were dried over anhydrous sodium sulfate and then stored in sealed glass vials. Obtained samples were evaluated for their essential oil components by GC/MS instrument (Agilent 6890 N GC and Agilent 5973 mass selective detector operating in the EI mode, USA)^37^.

### Statistical analysis

All obtained data analysis performed by SAS software and the means of each treatment were analyzed by Duncan’s multiple range test at the 95% level of probability (SAS Institute Inc., ver. 9.1, Cary, NC, USA).

## Results and Discussion

### Characterization of TiO_2_ NPs

**Figure 1:**
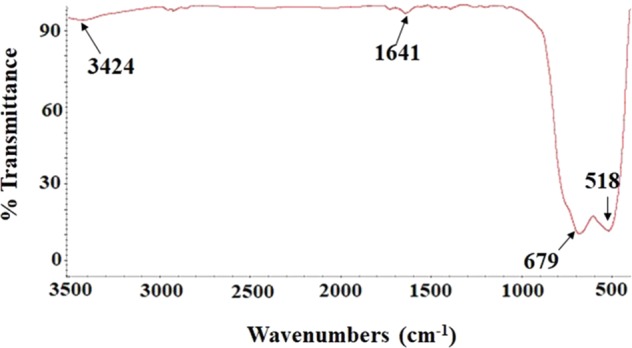
FTIR spectrum of TiO_2_ NPs.

FTIR spectrum was used for the chemical elucidation of the synthesized TiO_2_ NPs. The existence of unresolved stretching vibrations of Ti-O-Ti could be assigned as broad band in the region of 400–900 cm^−1^ (Fig. 1). In addition, two bands at 1620 and 3427 cm^−1^ were related to bending and stretching vibrations of O-H groups^38^.

**Figure 2:**
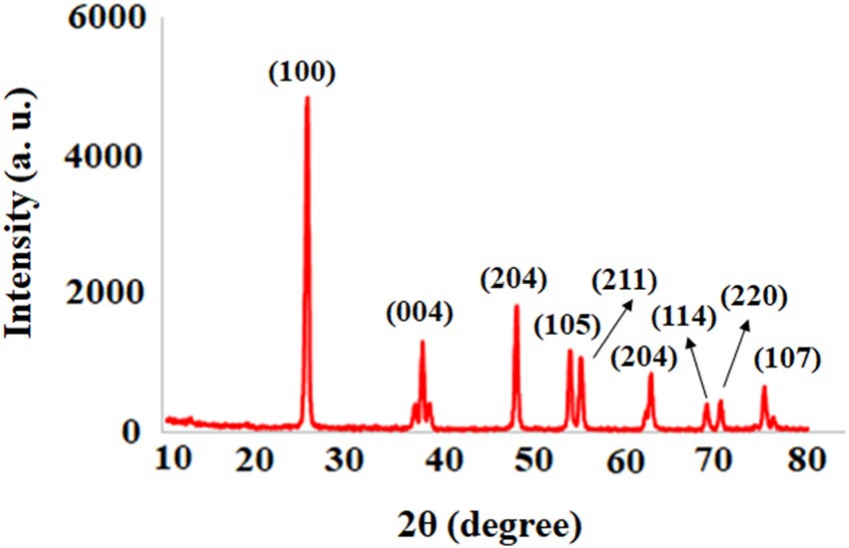
X-ray diffraction (XRD) pattern for TiO_2_ NPs.

X-ray diffraction (XRD) pattern of TiO_2_ NPs was investigated to study the structure and phase formation of the sample. According to Fig. 2, a well-crystallized anatase profile was observed for TiO_2_ NPs, in good agreement with the JCPDS data (JCPDS data file No. 21–1272).

**Figure 3:**
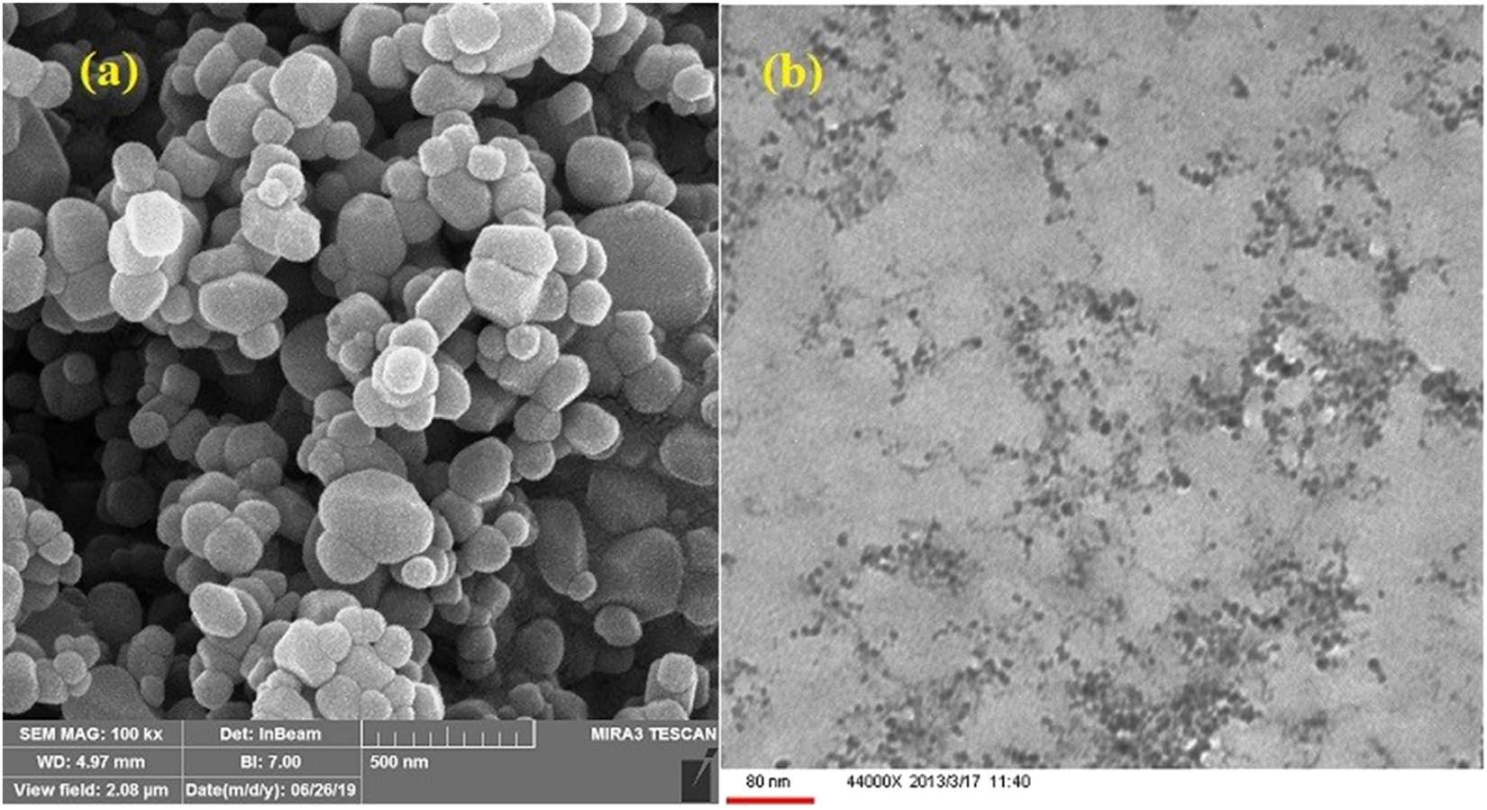
(**a**) SEM and (**b**) TEM images of TiO_2_ NPs.

Surface, size and the particle morphology of TiO_2_ NPs were imaged by scanning electron microscopy (SEM) and transmission electron microscopy (TEM) (Fig. 3a,b). Based on the SEM image, spherical-like shapes with particle diameter of 70–90 nm could be seen for the synthesized TiO_2_ NPs, while particle size was determined as 20–30 nm according to TEM. The difference in the size of nanoparticles obtained by SEM and TEM techniques may be related to the loss of stability of nanoparticles during the freezing-drying process as well as due to particle aggregation phenomena.

### Epifluorescence microscopy

**Figure 4:**
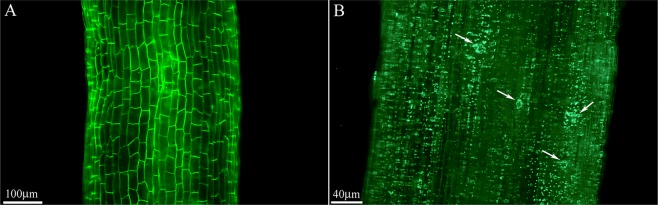
Epifluorescence microscopic images of *D. moldavica* L roots in 0 mg L^−1^ (**A**) and 200 mg L^−1^ (**B**) of TiO_2_ suspensions grown under control conditions.

Epifluorescence microscopy confirmed the uptake of different concentration of TiO_2_ NPs into Moldavian balm (*Dracocephalum moldavica* L.) root tissue (Fig. 4).

In the plants treated with high concentration of TiO_2_ (200 mg L^−1^), the presence of NP aggregates was indicated by fluorescent light spots inside the root (Fig. 4B). No spots were observed in control (0 mg L^−1^ TiO_2_) plants, as expected (Fig. 4A). Only a few studies exist on subcellular localization of TiO_2_ in plants. Present results demonstrated that the high concentration of TiO_2_ NPs increased their aggregations in the plant root. TiO_2_ was actively taken up in *Spirodela polyrrhiza* roots and aggregated in the plant cells at toxic concentration^39^ (See Supporting Information Fig. S1). Both studies demonstrated the entry of TiO_2_ NPs in the roots by markedly shiny spots at high inside the roots, representing aggregation. Similar to our observations, fluorescence microscopy imaging techniques were used to indicate the entrance of magnetic NPs into soybean plant tissues as shown in previous reports^26,39^. From an application point of view, various parameters such as size, concentration and aggregation of NPs are the most important issues in agriculture, playing important roles in determining reactivity, toxicity, fate, transport and risk in the environment^40^.

### Assessments of agronomic parameters

Plant agronomic parameters was significantly influenced by application of TiO_2_ NPs, salt stress and their interactions (Table 1).

**Table 1:** Effect of different concentrations of TiO_2_ NPs on key agronomic parameters of *D. moldavica* L. plants under salinity stress.

Salt stress × TiO_2_ (interaction effect)		Plant height (cm)	Shoot FW (g)	Shoot DW (g)	Leaf number	Leaf FW (g)	Leaf DW (g)
**Traits**							
0 mM NaCl	0 mg L^−1^ TiO_2_	44.33^b^	15.44^c^	4.72^b^	84.00^c^	4.21^c^	2.39^c^
	50 mg L^−1^ TiO_2_	45.00^b^	16.24^b^	5.10^a^	95.33^b^	4.72^b^	2.81^b^
	100 mg L^−1^ TiO_2_	62.33^a^	17.49^a^	4.69^b^	101.33^a^	5.34^a^	3.15^a^
	200 mg L^−1^ TiO_2_	40.00^de^	14.53^d^	3.83^c^	71.33^d^	3.70^d^	2.33^c^
50 mM NaCl	0 mg L^−1^ TiO_2_	33.66^hi^	8.10^h^	2.11^f^	44.33^g^	2.26^f^	0.69^h–g^
	50 mg L^−1^ TiO_2_	36.66^gf^	8.87^g^	2.43^e^	42.00^g^	3.57^d^	1.55^d^
	100 mg L^−1^ TiO_2_	43.00^bc^	10.48^e^	2.75^d^	57.33^e^	3.74^d^	1.58^d^
	200 mg L^−1^ TiO_2_	41.66^cd^	9.66^ef^	2.66^d^	51.66^f^	3.11^e^	0.58^hg^
100 mM NaCl	0 mg L^−1^ TiO_2_	30.00^j^	7.82^h^	0.63^i^	37.33^h^	2.27^f^	0.54^h^
	50 mg L^−1^ TiO_2_	32.00^ji^	7.65^h^	1.07^h^	40.66^gh^	1.88^g^	0.72^fg^
	100 mg L^−1^ TiO_2_	35.00^gh^	8.89^g^	1.95^g^	41.00^gh^	3.19^e^	0.76^f^
	200 mg L^−1^ TiO_2_	38.66^ef^	9.74^f^	2.17^f^	44.00^g^	3.63^d^	1.06^e^

^*^Different letters indicate significant differences at 5% level of confidence according to Duncan’s test.

Results demonstrated that the maximum plant height (≈62.33 cm) was observed in 100 mg L^−1^ TiO_2_-treated plants under control conditions. On the contrary, the lowest height was achieved in 100 mM NaCl without TiO_2_ treatment. Regarding shoot fresh weight, the maximum and minimum values were recorded in 100 mg L^−1^ TiO_2_-treated plants under no salinity and 50 mg L^−1^ TiO_2_ under 100 mM salinity conditions, respectively. In the case of shoot dry weight, 50 mg L^−1^ TiO_2_ NPs under control conditions demonstrated the highest value, whereas 100 mM NaCl resulted in the lowest value. Application of 100 mg L^−1^ TiO_2_ under no salinity conditions caused maximum leaf number (≈101.33), while 100 mM NaCl with no TiO_2_ application had the lowest (≈37.33). Current results also showed that the highest and lowest amounts of leaf FW were achieved in plants treated with 100 mg L^−1^ TiO_2_ without salinity and 50 mg L^−1^ TiO_2_ under 100 mM salinity conditions, respectively. Furthermore, plants treated with 100 mg L^−1^ TiO_2_ NPs without salinity stress had the highest leaf DW (≈3.15 g), as expected considering their FW. Lowest DW values were recorded in 100 mM NaCl-treated plants. In total, plants treated with 100 mg L^−1^ TiO_2_ displayed optimal performance for most agronomic traits, whereas worst-performing plants were the ones grown under severe salinity stress (100 mM NaCl). It is worth stating that TiO_2_ application, especially in low and medium concentrations, also improved agronomic parameters under control conditions, thus rendering them as potential growth promoters. Contrarily, plants treated with 200 mg L^−1^ TiO_2_ showed significant decrease in their agronomic attributes, indicative of toxicity effects. In addition, application of TiO_2_ NPs showed positive effects on the agronomic traits under salinity conditions and significantly ameliorated the stressor’s negative effects. In detail, approximately all TiO_2_ concentrations could reverse the negative effects of salinity stress by improving the agronomic parameters examined under different salinity levels; 100 mg L^−1^ TiO_2_ under 50 mM NaCl and 200 mg L^−1^ TiO_2_ under 100 mM NaCl achieved optimal performance in this regard.

Salt stress (NaCl) reduces plant growth due to its negative effect on photosynthesis rate, cell division and elongation, changes in enzymatic activity (subsequently affects protein synthesis), decrease in carbohydrates and growth hormone levels and disruption of biological and metabolic activities that finally could lead to growth inhibition^8^. Thus, plant height commonly decreases by increase in NaCl levels due to its destructive effects. Aziz *et al*.^41^ previously reported a reduction in plant height by increasing salinity levels. Considering the result of TiO_2_ application, all concentrations and 100 mg L^−1^ in particular, caused a significant increase in plant height, demonstrating that the application of TiO_2_ NPs ameliorated the negative effects of salinity. The observed decrease in leaf number and FW in moderate and high salinity levels is attributed to a reduction in cell expansion due to low turgor controlled by cellular water uptake and cell-wall extension^42^. In addition, Kapoor and Pande^43^ concluded that leaf numbers decreased under salinity conditions due to a reduction in branches per plant as a result of decrease in nutrient concentrations. Decline in fresh and dry weights, leaf numbers and abscissions under salinity stress were previously reported^44^. However, the positive effects of TiO_2_ NPs were observed in leaf numbers as well as fresh and dry weights in the current study. In this regard, Rahneshan *et al*.^45^ reported that TiO_2_ application enhanced absorption rate of macro- and micro-nutrients, improved plant growth characteristics (e.g., plant height, leaf number) and reduced negative effects of salinity by affecting photosynthesis and absorption of essential elements.

### Photosynthetic pigments

Application of TiO_2_ NPs had significant effects on photosynthesis pigments (Table 2).

**Table 2:** Effect of different concentrations of TiO_2_ NPs on photosynthesis pigments of *D. moldavica* L. plants under salinity stress.

Salt stress × TiO_2_ (interaction effect)		Chlorophyll *a* (mg g^−1^ FW)	Chlorophyll *b* (mg g^−1^ FW)	Carotenoids (mg g^−1^ FW)
0 mM NaCl	0 mg L^−1^ TiO_2_	3.19^d^	2.18^b^	0.65^bc^
	50 mg L^−1^ TiO_2_	4.47^c^	1.77^c^	0.73^ab^
	100 mg L^−1^ TiO_2_	5.38^a^	2.66^a^	0.81^a^
	200 mg L^−1^ TiO_2_	3.47c	1.37^c^	0.46^df^
50 mM NaCl	0 mg L^−1^ TiO_2_	2.4^fg^	0.69^fh^	0.52^ce^
	50 mg L^−1^ TiO_2_	2.62^f^	0.81^efg^	0.53^cd^
	100 mg L^−1^ TiO_2_	2.53^f^	1.37^d^	0.62^bc^
	200 mg L^−1^ TiO_2_	2.26^g^	0.58^h^	0.27^g^
100 mM NaCl	0 mg L^−1^ TiO_2_	1.46^j^	0.86^ef^	0.22 ^g^
	50 mg L^−1^ TiO_2_	1.99^h^	0.93^e^	0.21^g^
	100 mg L^−1^ TiO_2_	2.91^e^	0.68f^gh^	0.35^eg^
	200 mg L^−1^ TiO_2_	1.77^i^	0.6^gh^	0.31^fg^

^*^Different letters indicate significant differences at 5% level of confidence according to Duncan’s test.

The highest contents of chl *a*, *b* and carotenoids were observed in 100 mg L^−1^ TiO_2_ without salt stress. Furthermore, salinity stress decreased pigment content, but application of 100 mg L^−1^ TiO_2_ increased chl *a*, *b* and carotenoid contents under both salinity levels. 200 mg L^−1^ TiO_2_ led to significantly lower pigment contents compared with lower TiO_2_ concentrations similar to salt-stressed samples, suggesting toxicity. The observed decrease in photosynthesis pigment content under salt stress conditions could be attributed to reduced biosynthesis or more likely increased breakdown due to ROS damage of the pigments in cells, functional disorders observed during stomatal movement and instability of the pigment protein complex under salinity stress. Salinity stress is known to result in pigment breakdown due to accumulation of toxic ions in chloroplasts and ROS-induced oxidative stress in plants^45^. In addition, Hernandez *et al*.^46^ stated that pigment reduction in NaCl-sensitive plants happened due to changes in number and size of chloroplasts, starch content, disorganized chloroplast membranes, loss of envelope and disorganization of grana and thylakoids.

### Chlorophyll fluorescence

**Figure 5:**
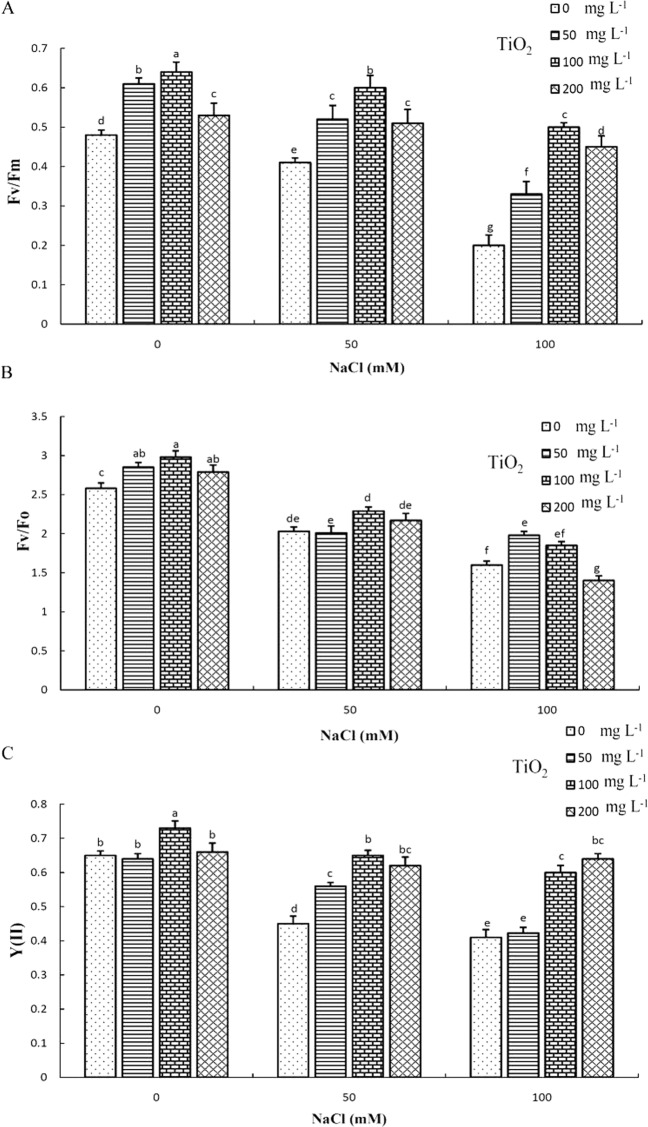
Effect of different concentrations of TiO_2_ NPs on chlorophyll florescence Fv/Fm (**A**), Fv/Fo (**B**), and Y(II) (**C**) of *D. moldavica* L. under salinity stress. Different letters indicate significantly different values at p < 0.05.

Moderate and high salinity levels significantly decreased chlorophyll fluorescence parameters including ^Fv^/_Fm_ (a ratio that indicates about the quantum efficiency of photosystem II: maximal quantum yield of PSII), ^Fv^/_Fo_ (a parameter that accounts for the simultaneous variations in Fm and Fo in determinations of the maximum quantum yield of PS II: Efficiency of the water-splitting complex on the donor side of PSII) and Y(II) (the complementary quantum yields of PS II). TiO_2_ NP application ameliorated the salt-induced drop in chlorophyll fluorescence parameters. Specifically, all TiO_2_ NP treatments increased ^Fv^/_Fm_ values under both control and stress conditions with highest values recorded following100 mg L^−1^ TiO_2_ NP application under control conditions (Fig. 5A). Furthermore, all TiO_2_ NP concentrations increased ^Fv^/_Fo_ with the highest value being recorded at 100 mg L^−1^ under control conditions, while NP pre-treatment ameliorated decreases recorded in this parameter under salt stress conditions (Fig. 5B). Similar findings were observed for Y (II) parameter, where NP application increased Y (II) under control conditions and reversed decreases observed in salt-stressed plants (Fig. 5C).

The significant decrease in these parameters was likely due to the dissipation of a major proportion of light energy as heat under salt stress^47^. Similar reduction in chlorophyll fluorescence parameters under salt stress was previously reported in maize^48^, as well as in sorghum^49^. Increase in the examined chlorophyll fluorescence parameters after TiO_2_ NP application could be attributed to enhancement in light energy of PSI absorbed by chloroplast membrane to be transferred to PSII, promotion of light energy conversion to electron energy and electron transport and acceleration of water photolysis and oxygen evolution^50^. In addition, Rubisco enzyme activity increased after TiO_2_ NP application due to increase in the expression of its mRNA^51^. Rubisco enzyme plays an important role in photosynthesis and optimal expression of this enzyme improves chlorophyll fluorescence parameters, while also increasing absorption of carbon dioxide in plants^52^. Overall, current findings suggest that TiO_2_ NPs potentially ameliorated the negative effects of salinity stress through the improvement in chlorophyll fluorescence parameters and by maximizing PSII efficiency^46^.

### Assessment of biochemical traits

#### Evaluation of hydrogen peroxide (H_2_O_2_) content

**Figure 6:**
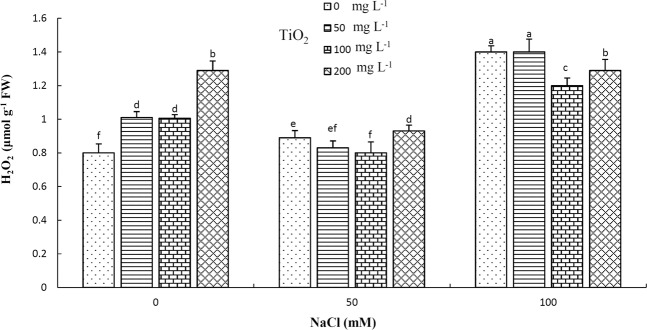
Effect of different concentrations of TiO_2_ NPs on H_2_O_2_ concentration of *D. moldavica* L. under salinity stress. Different letters indicate significantly different values at p < 0.05.

The highest (≈1.4 μmol g^−1^ FW) H_2_O_2_ content was observed in 100 mM NaCl-treated plants without TiO_2_ application, whereas control plants had the lowest content, along with plants treated with 50 and 100 mg L^−1^ TiO_2_ under 50 mM NaCl (Fig. 6).

In addition, application of 100 mg L^−1^ TiO_2_ in plants growing under moderate and high salinity stress as well as in 50 mg L^−1^ TiO_2_-treated plants under moderate NaCl stress decreased H_2_O_2_ content in leaf tissues compared with plants subjected to similar stress conditions without any TiO_2_ treatments. H_2_O_2_, produced in various vital processes of different organs cells, is highly toxic for cells and causes oxidative stress at high concentrations^53^, as well as damages to biological membranes via their peroxidation. Thus, the mentioned TiO_2_ treatments could amplify plant performance under saline conditions likely by decreasing oxidative stress and lessening membrane damage. Superoxide dismutase (SOD), as the primary ROS scavenger localizing in chloroplasts, mitochondria, peroxisomes and cytosol, catalyzes the disproportion of two O_2_·^−^ radicals to H_2_O_2_ and O_2_^54^. Moreover, H_2_O_2_ is scavenged by ascorbate-peroxidase (APX) in ascorbate-glutathione cycle and through guaiacol peroxidase (GP) and catalase (CAT) in cytoplasm and divided into water and oxygen^55^. The increased activity of the mentioned enzymes by TiO_2_ treatments in the present study might be another reason for the observed decrease in H_2_O_2_ values under salinity stress compared with control conditions. Although all TiO_2_ treatments increased H_2_O_2_ values and the high concentration of TiO_2_ might be considered as toxic, these increases were lower than those under salinity stress, demonstrating lower negative effects of NP treatments even at high concentration compared with salinity. Moreover, considering the positive impact of TiO_2_ towards lowering H_2_O_2_ content under salinity, NP treatments could be considered as beneficial for removing undesirable effects of salinity.

#### Evaluation of antioxidant enzymes

**Figure 7:**
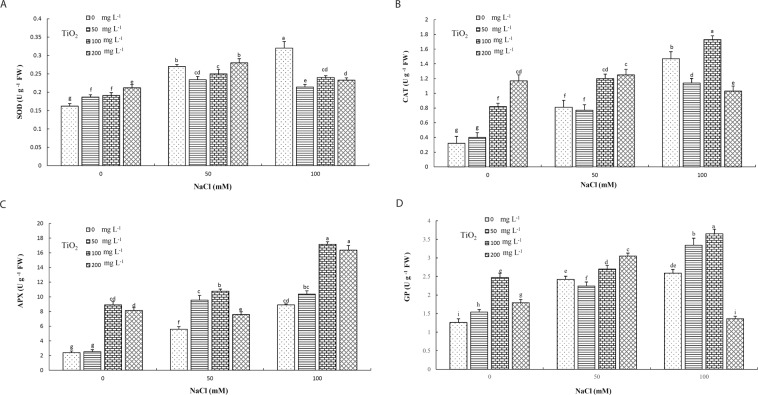
Effect of different concentrations of TiO_2_ NPs on SOD (**A**), CAT (**B**), APX (**C**) and GP (**D**) enzyme activity of *D. moldavica* L. under salinity stress. Different letters indicate significantly different values at p < 0.05.

Application of TiO_2_ NPs, salt stress and their interactions significantly affected superoxide dismutase (SOD) activity. The maximum and minimum activities were recorded in 100 mM NaCl-treated plants under no TiO_2_ application and control samples, respectively. SOD activity of leaf tissues under moderate and high salinity stresses increased significantly compared with controls. Amongst treatments, the highest activity was observed in 200 mg L^−1^ TiO_2_ under 50 mM NaCl, while the lowest was observed in 50 mg L^−1^ TiO_2_ under no salinity and 200 mg L^−1^ TiO_2_ under 100 mM NaCl (Fig. 7A).

In regard with catalase (CAT), enzymatic activity in leaf tissues under 50 and 100 mM NaCl increased significantly compared with control. Therefore, a positive regulation of CAT activity by salt concentration was observed; increasing NaCl levels resulted in increasing CAT activity, similar to SOD. The highest activity among treatments was achieved in 100 mg L^−1^ TiO_2_ under 100 mM NaCl, while the lowest was in 50 mg L^−1^ TiO_2_-treated plants under no salt stress. Considering CAT activity, TiO_2_ at 100 mg L^−1^ concentration generally increased enzymatic activity under both stress conditions compared with those plants at similar conditions without receiving any TiO_2_ treatment (Fig. 7B).

The highest and lowest ascorbate peroxidase (APX) activities were observed in 100 and 200 mg L^−1^ TiO_2_-treated plants under 100 mM NaCl and the control and 50 mg L^−1^ TiO_2_ under no salinity stress, respectively. Similar to SOD and CAT, increasing salinity levels lead to increasing APX activity, under no TiO_2_ treatment. TiO_2_ treatments increased APX activity under both non-stress and stress conditions, with these increases being higher than non-treated plants at the same conditions (Fig. 7C).

Maximum guaiacol peroxidase (GP) activity was observed in 100 mg L^−1^ TiO_2_ under 100 mM NaCl. In this regard, minimum activity was noticed in the control and 200 mg L^−1^ TiO_2_-treated samples under 100 mM NaCl. GP activity in leaf tissues under moderate and high salinity stress levels increased significantly compared with control samples. In fact, increase in salinity level increased GP activity. As well, TiO_2_ treatments increased the activity in which TiO_2_-treated plants had higher activity that non-treated ones under both non-stress and stress conditions (Fig. 7D).

In total, the activity of GP, APX, CAT and SOD significantly increased under both moderate and high salinity levels. In addition, TiO_2_ treatments at 100 and 200 mg L^−1^ concentrations increased antioxidant enzyme activities under control conditions. A similar increasing trend was observed in 50 and 100 mg L^−1^ TiO_2_-treated plants under both salinity levels for the above-mentioned enzymes. It is noteworthy that, although the applied salt stress increased enzymatic activities, highest levels were observed in 100 mg L^−1^-treated plants for CAT, APX and GP. SOD enzyme was an interesting exception as its activity was enhanced by TiO_2_ application under control conditions, whereas SOD activity decreased significantly in TiO_2-_treated plants under moderate and severe salt stress compared with plants without any TiO_2_ application. Additionally, the high concentration of TiO_2_ (200 mg L^−1^) applied in plants, under both salinity levels, showed lowest antioxidant enzymatic activity levels overall in comparison with plants treated with 50 and 100 mg L^−1^ TiO_2_ which could be correlated with toxicity phenomena.

Overall, it could be concluded that 100 mg L^−1^ TiO_2_ application under moderate and high salt stress induces antioxidant enzyme activities, thus contributing in the effective protection of plants from salinity. This is likely through the detoxification of ROS, which is known to over accumulate in saline environments^56^. ROS compounds are generated by normal cellular activities (e.g., fatty acids β-oxidation), photorespiration and biotic or abiotic stress conditions. ROS elimination is mainly achieved by antioxidant mechanisms such as antioxidant enzymes (e.g., SOD, CAT, APX)^57^. SOD is the key enzyme for neutralizing ROS as the first line of defense mechanism against oxidative stress. Enhancement in SOD activity is tightlylinked with increased protection against negative effects of stress factors^58^. CAT, another important antioxidant enzyme, scavenges H_2_O_2_ by converting it to water in peroxisomes and neutralizes its deleterious damages^59^. APX activity, yet another key antioxidant enzyme, eliminates H_2_O_2_ activity and modulates its steady-state level in various subcellular compartments of plants^60^. Moreover, high levels of intercellular H_2_O_2_ are known to induce cytosolic APX activity under salinity stress^61^. Thus, APX plays an important role in the collection and decomposition of H_2_O_2_ during stress^62^. GP acts as an electron transmitter to H_2_O_2_, in an attempt to detoxify cells under stress conditions by converting H_2_O_2_ into water^63^. Previous studies reported considerable induction of enzymatic antioxidants under salinity stress, thus preventing ROS-related damage (e.g. Filippou *et al*.^7^). Our findings are in agreement with Weisany *et al*.^56^, who noted that CAT and APX enzymatic activities in soybean increased under salinity stress due to oxidative reactions caused by higher levels of H_2_O_2_. Regarding the enhancement in antioxidant enzyme activities of the plants treated with TiO_2_ NPs under salt stress, positive interactions might take place which likely provide better signaling towards the activation of these defense enzymes. Moreover, the observed increases in SOD, CAT, APX and GP enzymatic activities under salinity in the present study might be related with the high intercellular H_2_O_2_ levels in Moldavian balm leaf tissues. Likewise, enhancement in SOD, CAT, APX and GP activities was observed in plants treated with TiO_2_ NPs. In addition, the lowest H_2_O_2_ content was observed in 100 mg L^−1^ TiO_2_-treated plant. Therefore, it could be concluded that the lowest H_2_O_2_ content recorded after 100 mg L^−1^ TiO_2_ application was closely related to the significantly increased activities of CAT, APX and GP in the same samples. ROS detoxification after TiO_2_ NP application might be due to stabilized composition of cells and improved physical properties of cell membranes. Lei *et al*.^11^ reported that application of TiO_2_ NPs under drought stress increased antioxidant enzyme activities in plants due to a reduction in lipid peroxidation and improvement in membrane integrity.

### Essential oil content and composition

**Figure 8:**
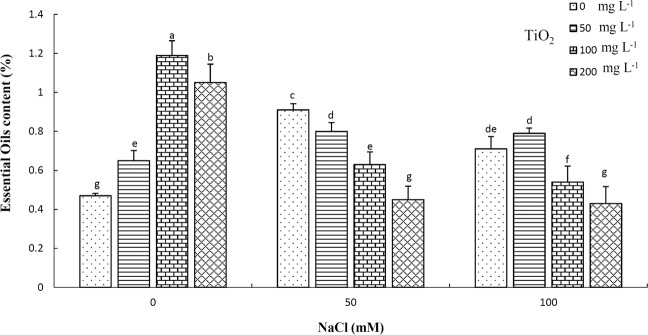
Effect of different concentrations of TiO_2_ NPs in essential oil content (%) of *D. moldavica* L. under salinity stress. Different letters indicate significantly different values at p < 0.05.

Essential oil content was significantly affected by salinity, TiO_2_ and their interactions. The highest essential oil content (≈1.19%) was recorded in 100 mg L^−1^ TiO_2_-treated plants under no salinity stress. The lowest (≈0.43%) contents were observed in 200 mg L^−1^ TiO_2_ under 50 and 100 mM NaCl, as well as in control samples (Fig. 8). Salinity stress positively affected essential oil content. Generally, both salinity levels increased essential oil content, but maximal yield was achieved by 50 mM NaCl. Similarly, TiO_2_ NP application had a positive impact in essential oil content, significantly increasing it under control conditions with optimal content recorded following 100 mg L^−1^ TiO_2_ NP application. However, under salinity conditions, TiO_2_ treatments had no considerable impact on this component compared with non-treated plants under stress.

The essential oil composition of *D. moldavica* L. under different salt stresses and TiO_2_ NPs applications is shown in Table 3. Base on the results, 29 constituents were identified by GC/MS analysis. Main components were geranial, z-citral, geranyl acetate and geraniol. 50 mM NaCl caused significant decrease in geranial and z-citral as well as minor decrease in geraniol, while it significantly increased geranyl acetate concentration. However, 100 mM NaCl had a different effect, as geranial concentration was not affected, geraniol and geranyl acetate content showed increase, while z-citral decreased compared with control samples.

**Table 3:** Effect of different concentrations of TiO_2_ NPs on essential oil composition of *D. moldavica* L.

	Compounds	RI	0 mg L^−1^ TiO_2_			50 mg L^−1^ TiO_2_			100 mg L^−1^ TiO_2_			200 mg L^−1^ TiO_2_		
			0 mM NaCl	50 mM NaCl	100 mM NaCl	0 mM NaCl	50 mM NaCl	100 mM NaCl	0 mM NaCl	50 mM NaCl	100 mM NaCl	0 mM NaCl	50 mM NaCl	100 mM NaCl
1	Camphene	946	—	—	—	0.19	—	0.19	0.22	0.21	0.22	0.2	—	—
2	Sabinene	969	—	—	0.35	0.35	2.07	0.37	0.6	0.35	0.35	0.31	0.2	0.43
3	1,8-Cineole	1026	0.26	0.73	0.73	0.15	—	1.28	0.23	0.35	0.82	0.2	0.44	0.65
4	Fenchone	1083	0.05	—	—	0.05	—	0.14	0.06	0.07	—	0.06	—	—
5	Linalool	1095	0.47	0.49	0.5	0.36	—	0.67	0.59	0.53	0.71	0.5	0.44	0.62
6	Pinocarveol	1135	0.18	0.19	—	0.19	—	0.26	0.16	0. 2	0.22	0.19	—	—
7	Camphor	1141	0.87	0.59	0.59	0.78	—	0.74	1.03	0.88	0.88	0.85	0.59	0.78
9	Borneol	1165	—	—	—	0.09	—	—	0.07	0.07	—	0.08	—	—
10	Menthol	1167	1.31	1.01	1.01	1.19	1.12	1.4	1.57	1.21	1.46	1.31	0.99	1.36
11	beta fenchyl alcohol	1180	0.16	—	—	0.66	—	1.04	0.2	0.38	0.41	0.29	—	—
12	Myrtenol	1194	0.09	1.2	0.25	0.08	4.36	0.8	0.07	0.84	2.37	0.07	—	—
13	n-Dodecane	1200	—	—	—	0.06	—	0.14	0.06	0.06	—	0.05	—	—
14	Nerol	1227	0.26	0.34	0.42	0.23	—	0.55	0.35	0.32	0.35	0.23	0.28	0.36
15	Z-citral	1238	25.26	18.53	23.62	26.71	18.13	21.96	26.98	22.13	20/1	23.5	20.7	23.2
16	Geraniol	1252	5.33	4.4	6.72	4.37	5.66	5.28	7.51	5.84	5.64	4.94	6.4	6.21
17	Geranial (E-citral)	1267	41.88	36.77	41.91	44.05	32.98	39.1	43.97	39.85	40.65	40.56	41.72	38.76
18	Carvacrol	1298	—	—	—	0.03	—	—	0.1	—	—	0.1	—	—
19	Methyl geranate	1322	0.24	0.23	0.27	0.26	—	0.21	0.21	1.21	1.21	0.19	0.24	0.28
20	Neryl acetate	1361	0.57	0.92	0.86	0.56	—	0.78	0.47	0.75	0.68	0.64	0.88	0.86
21	Geranyl acetate	1381	18.96	22.01	19.99	15.09	15.91	16.92	11.17	18.65	16.5	19.27	23	21.1
22	n-Tetradecane	1400	0.12	0.15	—	0.12	—	0.11	0.15	0.2	—	0.1	—	0.28
23	(E)-β -caryophylene	1417	0.12	0.78	—	0.13	1.35	0.21	0.14	0.23	—	0.22	—	—
24	Germacrene D	1484	0.06	1.35	—	0.4	2.39	0.4	0.45	0.97	0.69	0.09	0.5	0.91
25	β-selinene	1489	0.13	0.49	—	0.12	—	0.1	0.15	0.18	0.18	0.1	0.21	—
26	Spathulenol	1577	0.15	0.27	—	0.14	—	0.14	0.16	—	—	0.19	0.24	—
27	Caryophyllene oxide	1582	0.05	—	—	0.07	—	—	—	—	—	0.06	—	—
28	β-eudesmol	1649	0.09	2.05	0.31	0.1	0.23	0.37	0.11	0.12	—	0.12	0.25	—
29	Bisabolol oxide	1656	—	—	—	—	—	—	—	0.08	—	0.07	—	—

Under prolonged salinity stress. RI values represent retention indices determined on GC/MS capillary column.

TiO_2_ application at 50 and 100 mg L^−1^ concentrations under control conditions enhanced geranial and z-citral content, while 100 mg L^−1^ increased geraniol content. Contrarily, these TiO_2_ treatments under both salinity levels decreased geranial and z-citral content with the highest decrease being recorded at 50 mg L^−1^ TiO_2_ application under 50 mM NaCl stress. Moreover, geranyl acetate was significantly decreased at the above-mentioned TiO_2_ treatments under both stress and non-stress conditions with the exception of 100 mg L^−1^ TiO_2_ under 50 mM NaCl. 200 mg L^−1^ TiO_2_ treatment demonstrated no difference in geranial, z-citral, and geraniol values under non-stress condition. In addition, this treatment caused a decrease in z-citral and increase in geraniol content under both salinity levels. Geranial showed no significant difference under 50 mM salinity, while it lowered under 100 mM NaCl. Moreover, geranyl acetate content increased significantly following 200 mg L^−1^ TiO_2_ treatment under both control and stress conditions with the highest increase being recorded at 50 mM NaCl application. Furthermore, in spite of considerable enhancement in myrtenol and germacrene D contents by 50 mM NaCl without TiO_2_ application, their highest values were observed in 50 mg L^−1^ TiO_2_ under 50 mM NaCl stress. Regarding nerol content, although salinity increased its content (increasing NaCl concentrations leading to increasing nerol content), the highest value was observed in 50 mg L^−1^ TiO_2_-treated plants under 100 mM NaCl stress. Nerol content was also increased following 100 mg L^−1^ TiO_2_ under both control and stress conditions as well as following 200 mg L^−1^ TiO_2_ under 100 mM NaCl.

Essential oils of Moldavian balm, as an important aromatic and medicinal plant, have various application in the pharmaceutical industry. Considering the importance of its essential oils, any treatment with positive effects on its essential oil content and dominant constituents could be of great value to growers. The positive effect of TiO_2_ NPs was previously reported in *Salvia officinalis* essential oil content and constituents^55^. Taking into account these factors, the current study examined the effect on Moldavian balm under normal and salt stress conditions. Present results revealed that the essential oil content increased under both NaCl levels, in agreement with Khalid and Teixeira de Silva^64^ and Neffati *et al*.^65^. However, a similar trend was not recorded for individual components of the essential oil profile, since the dominant constituents mostly decreased following salt stress particularly 50 mM NaCl. This decrease might be attributed to an impairment in photosynthesis, changes in metabolic systems and increase in osmotic pressure, which might then decrease nutrients and water uptake. Salinity stress has been previously shown to modify essential oil production and profile^41^. Current results demonstrated that salinity stress altered the content of specific essential oil components in *Dracocephalum moldavica* L. plants, in agreement with Khalid and Teixeira de Silva^64^ and Neffati *et al*.^65^ who attributed such changes to the regulation of the activity of essential oil biosynthetic enzymes following salt stress imposition.

TiO_2_ application caused a remarkable increase in essential oil content under control conditions with maximum content being observed at 100 mg L^−1^ concentration. Results were in accordance to those reported by Ahmad *et al*.^66^, who demonstrated that TiO_2_ NP application increased essential oil content in *Mentha piperita* L. Furthermore, Lafmejani *et al*.^67^ reported that Fe NPs foliar application increased essential oil content in *M. piperita* plants. Such an increase in essential oil content could be potentially explained by the observed increase in growth, photosynthesis, expression of secondary metabolite enzymes and size and distribution of oil glands as special sites for biosynthesizing essential oils following NP application^66^. In line with the increase in essential oil content, 100 mg L^−1^ TiO_2_ NP application increased main components of essential oil profile. This could be the result of increased expression of specific biosynthetic enzymes involved in the production of components and availability of substrates, in line with previous findings by Ahmad *et al*.^66^ and Lafmejani *et al*.^67^. The actual mechanism by which NP application modulates plant secondary metabolites is not yet fully elucidated. Recently, coordinated phytochemical and genomic studies confirmed that NPs might act as elicitors for secondary metabolite production in plants by inducing different cellular signal transduction pathways (e.g., mitogen-activated protein kinases, calcium flux and ROS metabolism). Accordingly, the observed changes in the above-mentioned pathways might lead to alterations in gene expression levels and metabolic enzyme activation that could alter secondary metabolite production^68^.

## Conclusion

Nanotechnology is a highly promising novel approach that has great potential for application towards plant protection against different stress conditions. TiO_2_, recently developed nanoparticles with profound effects in plant morphological, physiological and biochemical properties, could improve overall plant performance. Its application in Moldavian balm plants demonstrated these positive effects under moderate and severe salinity stress as enhanced agronomic traits under both control and stress conditions. TiO_2_ NP application additionally lowered H_2_O_2_ content and increased antioxidant enzyme activities), thus ameliorating oxidative damage and demonstrating positive effects in plants under both conditions. Importantly, enhancement in essential oil content by TiO_2_ treatments demonstrated another positive impact of TiO_2_ NPs with implications in the potential for commercial application Interestingly, application of high concentration of TiO_2_ (200 mg L^−1^) showed toxic symptoms in specific parameters, likely linked with NP aggregation in high concentrations which lead to increased ROS content. Consequently, TiO_2_ might act as an inducer of secondary metabolite production (such as essential oils) and trigger for the activation of the enzymatic defense system, ultimately enhancing plant performance under control and stress conditions and thus acting as a promising stress protecting and growth promoting molecule.

## Supplementary information

Supplementary Information.
