# Supplementary material for: Titanium dioxide nanoparticles (TiO2 NPs) promote growth and ameliorate salinity stress effects on essential oil profile and biochemical attributes of Dracocephalum moldavica
Source: Sci Rep. 2020 Jan 22;10:912. doi: 10.1038/s41598-020-57794-1 (PMC6976586; doi:10.1038/s41598-020-57794-1)
Supplement: Supplementary file 1 — Supplementary Information. [file 41598_2020_57794_MOESM1_ESM.docx]

**Titanium dioxide nanoparticles (TiO_2_ NPs) promote growth and ameliorate salinity stress effects on essential oil profile and biochemical attributes of *Dracocephalum moldavica***

Gholamreza Gohari^1*^, Asghar Mohammadi^1^, Ali Akbari^2 *^, Sima Panahirad^3^, Mohammad Reza Dadpour^3^, Vasileios Fotopoulos^4^ and Seisuke Kimura^5,6^

^1^ Department of Horticultural Sciences, Faculty of Agriculture, University of Maragheh, Maragheh, Iran

^2^ Solid Tumor Research Center, Cellular and Molecular Medicine Institute, Urmia University of Medical Sciences, Urmia, Iran

^3^ Department of Horticultural Sciences, Faculty of Agriculture, University of Tabriz, Tabriz, Iran

^4^Department of Agricultural Sciences, Biotechnology and Food Science; Cyprus University of Technology Limassol, Cyprus

^5^Department of Industrial Life Sciences, Kyoto Sangyo University, Kamigamo-Motoyama, Kita-Ku, Kyoto 603-8555, Japan

^6^ Center for Ecological Evolutionary Developmental Biology, Kyoto Sangyo University, Kamigamo-Motoyama, Kita-Ku, Kyoto 603-8555, Japan

*Corresponding author: [gohari.gh@maragheh.ac.ir](mailto:gohari.gh@maragheh.ac.ir)


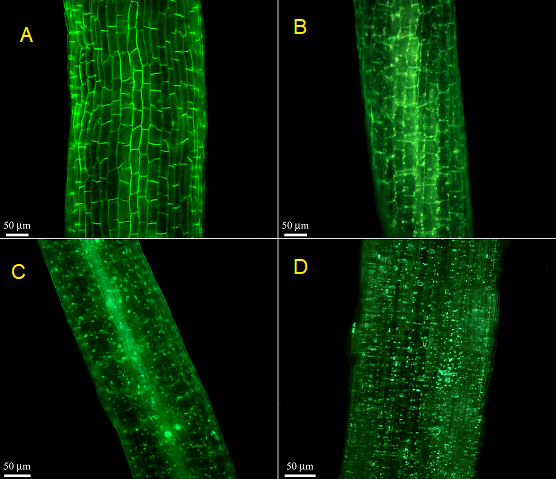


**Figure S1.** Epifluorescence microscopic images of *D. moldavica* L roots in 0 mg L^-1^ (A) and 50 mg L^-1^ (B), 100 mg L^-1^ (C), and 200 mg L^-1^ (D) of TiO_2_ suspensions grown under control conditions.
